# Transcriptional Responses to Alkaline pH Across Fungi: Common and Differential Features, and Biotechnological Applications

**DOI:** 10.3390/ijms262311450

**Published:** 2025-11-26

**Authors:** Joaquín Ariño

**Affiliations:** 1 Institut de Biotecnologia i Biomedicina, Universitat Autònoma de Barcelona, 08193 Cerdanyola del Vallès, Spain; joaquin.arino@uab.cat; 2 Departament de Bioquímica i Biologia Molecular, Universitat Autònoma de Barcelona, 08193 Cerdanyola del Vallès, Spain

**Keywords:** transcriptional responses, environmental alkalinization, gene regulation, fungi, biotechnological applications

## Abstract

The transcriptional response to alkalinization in *Saccharomyces cerevisiae*, *Aspergillus nidulans* and *Candida albicans* raised the interest of the scientific community many years ago for diverse reasons, and the underlying signaling pathways have been elucidated in these organisms in detail. Within the last few years, transcriptomic data for other fungal species have become available, although in most cases little is known about the molecular basis controlling their adaptive response. The objective of this work is to provide an overview on how different fungi remodel their gene expression in response to environmental alkalinization, highlighting the similitudes and differences among them. Microbial stress-responsive promoters have been considered useful tools for biotechnological applications, such as expression of recombinant proteins of industrial interest. Recent work, emphasizing the usefulness of alkaline pH-inducible promoters for heterologous protein production, will also be discussed.

## 1. Introduction

Most fungi grow preferentially at acidic pH, and switching to an alkaline environment involves a stress condition to which the organism responds, triggering a rapid transcriptional response. About 25 years ago, with the advent of technologies able to interrogate massively the mRNA levels of a cell or tissue, such a transcriptional response could be investigated at the genome-wide level in several fungal species. *S. cerevisiae* was among the first studied, likely as a result of the availability of its genomic sequence and its relevance as an industrial and research model organism, followed by *A. nidulans*, where antibiotic production is regulated by ambient pH [1], and by *C. albicans*, because its ability to adapt to environmental pH is fundamental to pathogenesis [2]. Since then, our understanding of the molecular details of the transcriptional responses in these fungi has largely increased, and genome-wide transcriptomic studies on other fungi have appeared. This review attempts to summarize what is in common and what is different in the profiles of mRNA changes invoked by shifting different fungi to an alkaline environment with the aim to extract general conclusions when possible. Eleven ascomycetes belonging to diverse phylogenetic branches and one basidiomycete (*Mycosarcoma maydis*, previously known as *Ustilago maydis*) will be examined (Table 1). As expected, the organisms investigated so far reflect specific biases: they either constitute well-stablished research models, such as *S. cerevisiae* or *S. pombe*, can be pathogenic (*C. albicans*, *A. fumigatus*), or have industrial interest (*S. cerevisiae*, *Aspergilllus niger*, *Komagataella phaffii*). Although mention will necessarily be made regarding pH sensing and signaling pathways involved in these responses, this review is not meant to replace previous works specifically focused on these aspects [3,4,5,6,7]. When available, I have retrieved and analyzed in a more general context the supplementary data provided with the publications and/or the original data deposited in public repositories. However, it must be noted that this review combines data obtained by two different methodologies—DNA microarrays and RNA sequencing—and that, even in the former case, the platforms used in the different reports are often different (dsDNA, short or long oligonucleotides, one- or two-dye labeling). Such heterogeneity adds a layer of difficulty when comparisons are made. More importantly, as will be discussed at the end of this review, sampling times after pH change range from a few minutes to 24 h among the different reports. Such differences imply that caution must be taken when attempting to extract direct conclusions by comparison of the reported data.

## 2. The Transcriptional Response to High pH Across Fungi

### 2.1. Saccharomyces cerevisiae

Stress responses in *S. cerevisiae* are heavily based on triggering a transcriptional response [8], and shifting yeast cells from the preferred acidic environment to an alkaline one is not an exception. The early identification of *ENA1* [9], *SHC1*, and *SCY1* [10] as alkaline pH-inducible genes was followed by the generation of a wealth of information mainly based on the use of DNA microarray analysis.

In a pioneering work, Lamb and coworkers [11] identified 71 genes induced after 2 h of growth at pH 8.0 in comparison with pH 4.0, highlighting some of the markers that would become typical for the alkaline response in this organism. Thus, the authors confirmed the induction of the Na^+^-ATPase *ENA1* by alkaline pH and reported the induction of several genes involved in phosphate homeostasis known to respond to phosphate starvation. These included those encoding the high-affinity H^+^/Pi transporter Pho84, the high-affinity Na^+^/Pi transporter Pho89, the secreted phosphatases Pho5 and Pho11, or Spl2, a protein that downregulates low-affinity phosphate transport during phosphate limitation. The set of induced genes also included *VTC1*/*NRF1*, *VTC3*/*PHM2*, and *VTC4*/*YJL012C*, at that time not yet recognized as components of the polyphosphate biosynthetic pathway [12,13]. This report [11] also highlighted several genes involved in adaptation to iron and copper starvation, such as the siderophore transporters *ARN3/SIT1* and *ARN4/ENB1*, the ferro-O_2_-oxidoreductase *FET3*, the low-affinity iron plasma membrane transporter *FET4*, ferric reductases *FRE1*, *FRE3*, *FRE4*, and the high-affinity copper transporter *CTR3*. The authors also observed induction of the gene *TIS11/CTH2*, later found crucial in the response to iron deficiency by specifically downregulating mRNAs encoding proteins that participate in many iron-dependent processes [14], and of the genes *YOR382W*/*FIT2* and *YOR383C*/*FIT3*, encoding mannoproteins involved in the retention of siderophore-iron in the cell wall [15]. Additionally, they found induced *YOL154W* (later named *ZPS1*), which responds to zinc starvation [16] and encodes the closest *S. cerevisiae* homolog of the alkaline-inducible *C. albicans* gene *PRA1*.

**Table 1:** Experiments described in this work.

Organism	Experimental Conditions	Technology	Reference
*Saccharomyces cerevisiae*			
	pH 6.0 to 7.9, time course 0–100 min, WT	Affymetrix YE6100 gene chips	[8]
	pH 4.0 to pH 8.0 (2 h), WT, *rim101* mutant	GeneFilter macroarray	[11]
	pH 6.4 to 7.6 (5, 25, 45 min), WT	DNA microarrays [17]	[18]
	pH 6.2 to 8.0, at 10, 20, and 45 min (WT, *cnb1*, *crz1* mutants)	DNA microarrays [17]	[19]
	pH 6.2 to pH 8.2, WT, and *slt2* mutant (15 and 30 min)	DNA microarrays [17]	[20]
	pH 5.5 to pH 8.0, WT and *msn2 msn4* mutant (10 and 30 min,)	DNA microarrays [17]	[21]
	pH 5.5 to pH 8.0/glucose starvation, *snf1* mutant, 10 min	DNA microarrays [17]	[22]
	Time course, pH 8.0, Genomic run-On, comparison with other stresses and mRNA instability	DNA microarrays [17]	[23]
*Aspergillus nidulans*			
	pH 4.0 to 8.0, WT and *sltA* mutant (1 h)	RNA-seq	[24]
*Aspergillus niger*			
	Growth at pH 2.5, 4.5 and 6.0	Affimetrix microarrays	[25]
*Aspergillus fumigatus*			
	pH 5.0 to 8.0, 1 h	Af293 DNA amplicon microarrays	[26]
	pH 5 to 8, time course (5, 15 30, 45, and 60 min)	TIGR *A. fumigatus* oligo slides v. 3	[27]
*Candida albicans*			
	WT and *rim101* mutant, pH 4.0 to pH 8.0, (4 h)	DNA microarrays	[28]
	pH 4 and 7.6 (several hours), WT, *rim101*−/− strain, and strain overexpressing *RIM101*	RNA-seq	[29]
*Aureobasidium pullulans*			
	Growth at pH 4, 7, and 10	RNA-seq	[30]
*Ustilago maydis*			
	pH 9.0 for 14 h, WT and *rim101* mutant	DNA microarrays NimbleGen	[31]
	pH 3.0, 7.0, and 9.0 for 16 h	DNA microarrays NimbleGen	[32]
*Trichoderma reesei*			
	pH 3, 4.5, and 6, after 17 and 24 h	DNA microarrays	[33]
*Trichoderma virens*			
	pH 4 to 8.0, pacC +/− (1 h)	DNA microarrays (oligos)	[34]
*Schizosaccharomyces pombe*			
	pH 5.4 to 8.0 (2 h)	DNA microarrays (3D-Gene *S. pombe* Yeast Oligo Chip 6k)	[35]
*Debaryomyces hansenii*			
	pH 6 to pH 8.0, +/− 1 M Na^+^ (3 h)	DNA microarrays	[36]
*Komagataella phaffii*			
	pH 5.5 to pH 8 8.2, WT strain on YPD and YPGly (15, 30, and 60 min)	RNA-seq	[37]
	pH 5.5 to pH 8, WT, *crz1* and *rim101* mutants (15, 30, and 60 min)	RNA-seq	[38]

Notes: WT, wild type; *Ustilago maydis* is currently designated as *Mycosarcoma maydis*.

The relevance of the transcription factor Rim101, homologous to PacC in *Aspergillus nidulans* (see below), in the alkaline response of specific genes was also investigated. It was found that induction of *ARN4* was blocked by deletion of *RIM101* and that of *ENA1* was partially attenuated, whereas the alkaline-induced expression of *PHO11/12*, *PHO84*, *CTR3*, *FRE1*, and *TIS11* was not affected by deletion of the transcription factor. This was a remarkable finding, since it showed that while Rim101 was indeed involved in the transcriptional response to alkalinization in *S. cerevisiae*, there were alternative alkali-sensitive signaling pathways. Because of technical issues, the authors did not report repressed genes in this work. In a subsequent work [39], the same laboratory identified 17 genes that were upregulated ≥ 2-fold and 18 genes that were downregulated in a *rim101*Δ strain compared with the wild-type (including several iron-related genes, such as *ARN1*, *ARN4*, and *FET3*). Importantly, in this work the authors showed that in contrast to the well-established function of PacC as a direct activator of alkaline pH-induced genes [40], in *S. cerevisiae*, Rim101 would act as a transcriptional repressor. Rim101-induced repression of Nrg1, a transcriptional repressor itself, would explain, in part, the induction of two alkaline pH responsive genes, *ENA1* and *ZPS1*. The role of Nrg1 in repressing *ENA1* expression (acting downstream of both Rim101 and Snf1) was further established a few years later by Platara and coworkers [41].

In parallel, in their comprehensive study on transcriptional responses to a variety of stresses, Causton and coworkers [8] investigated the response to alkaline pH (pH 6.0 to 7.9 transition) as a time course up to 100 min and set a threshold of 3-fold change to select for differentially expressed genes (DEGs). Analysis of the original data yields 427 induced and 279 repressed genes at least at one time-point. However, while in this work a few phosphate-related genes, such as *PHO89* and *SPL2*, as well as *ENA2* (likely *ENA1*) and *KHA1* (a putative K^+^/H^+^ antiporter localized to Golgi vesicles), and several genes involved in transition metal homeostasis (*TIS11*, *FET3*, *ARN3/SIT1*, *ARN4/ENB1 ZPS1*, and *FIT2*) were found induced, the overlap with Lamb’s study was relatively poor. Notably, *PHO11*, *PHO5*, and *PHO12* were found among the repressed genes. The set of induced genes was enriched in genes involved in glycogen (*GSY1*, *GSY2*, *GDB1*, and *GLC3*) and trehalose (*TPS1*, *TPS2*, *TPS3*, *TSL1*, *ATH1*, and *NTH1*) metabolism.

Shortly after, Serrano and coworkers evaluated the short-term response to mild alkalinization (pH 7.6) for 5, 25, and 45 min [18]. Under these conditions, they found 150 genes induced ≥ twofold at least at one time point, and 232 genes repressed. The data confirmed the impact of even mild alkalinization on *ENA1* expression and on phosphate metabolism, with induction of *PHO89*, *PHO84*, *PHO12*, *PHO11*, *VTC1*, *VTC3*, and *VTC4*, as well as numerous genes required for iron and copper homeostasis (*FRE1*, *FET3*, *FTH1*, *FIT2*, *ARN1*, *ARN2*, *ARN3*, and *CTR1*). Two years later, the same laboratory clarified the repeated presence of iron and copper-related genes in previous transcriptomic studies, by showing that the availability of iron and copper is a major limitation for growth of *S. cerevisiae* under alkaline pH conditions [42]. The authors also found signals of appearance of oxidative stress, with induction of *SOD1* (encoding superoxide dismutase), *GRX2* (glutaredoxin), *GRE2* (methylglyoxal reductase), and *TRR1* (thioredoxin reductase). Among the repressed ones, they reported multiple genes involved in purine biosynthesis and amino acid metabolism (including Arg, Lys, and His). Importantly, in this work, it was shown, by functional mapping of the *ENA1* promoter, that the induction by alkalinization of this Na^+^ ATPase was dependent not only on Rim101, but also on the presence of a functional calcineurin phosphatase pathway. In addition, the authors showed that *PHO84* and *PHO12* responses were unaffected in *crz1* cells (lacking the transcription factor acting downstream of calcineurin) but required the presence of Pho2 and Pho4, indicating that alkalinization was invoking a canonical phosphate starvation response. However, part of the alkali-induced expression of *PHO89* was maintained in *pho4* or *pho2* cells, but it was fully abolished in a *crz1* strain or in the presence of FK506 (a calcineurin inhibitor). Overall, these experiments provided evidence that signaling of alkaline pH in *S. cerevisiae* was a more complex process than expected.

The participation of the calcineurin pathway in the response to alkali was further explored by Viladevall and coworkers [19], who found that activation of calcineurin occurred in response to a sharp (seconds) and transient rise in cytoplasmic calcium from extracellular sources. DNA microarray analysis was performed as a time course (10, 20, and 45 min after switching cells to pH 8.0) in experiments including calcineurin-deficient (*cnb1*) and *crz1* mutants and revealed the induction (log2 ≥ 1.0) of 266 genes, 60% of them peaking after 10 min of alkalinization (early responsive genes). Exposure to alkali resulted in a decrease in mRNA levels of at least 2-fold for 157 genes, of which a substantial number (33 genes) corresponded to ribosomal proteins or to proteins related to ribosome assembly. These data confirmed the induction of *ENA1*, *KHA1*, *PHO89*, and *PHO84*, as well as several iron and copper homeostasis-related genes. Interestingly, several genes involved in glycogen (*GSY1*, *GSY2*, and *GLC3*) and trehalose (*TPS1*, *TPS2*) metabolism, not identified in the previously described Lamb and Serrano papers [11,18] were found among the induced genes. Similarly, this work included a significant number of hexose transporter (mostly high-affinity) encoding genes, such as *HXT2*, *HXT11*, *HXT7*, *HXT6*, *HXT10*, *HXT4*, and *HXT5*. It was found that induction of 10% of the alkali-sensitive genes was dependent, in full or in part, on *CNB1*, which encodes the regulatory subunit of the phosphatase calcineurin (and most of them required the presence of *CRZ1* as well). In general, Crz1-dependent genes were “early” genes, in agreement with the almost immediate rise in intracellular calcium [19] and the finding that alkalinization triggers rapid binding of Crz1 (1 to 5 min) to over 150 intergenic regions upon transferring *S. cerevisiae* cells to pH 8.0 [43]. Notably, the induction of oxidative response genes (*GRX1*, *TRX2*, *PRX1*, *GPX2*, *TRR1*, among others) was observed again, and this prompted the authors to confirm, by cellular staining, the induction of ROS production after 30 min of alkalinization. In this same work, they identified Yap1, a basic leucine zipper (bZIP) transcription factor required for oxidative stress tolerance, as necessary for high pH induction of *PRX1*, thus adding a further layer of complexity to the mechanisms of alkaline pH signaling.

Serrano and coworkers [20] examined the transcriptional response to more extreme pH (8.2) by collecting samples 15 and 30 min after the pH shift. The aim was to explore the contribution of the Pkc1-Slt2 cell wall integrity (CWI) pathway to alkaline tolerance, a notion based on the sensitivity to high pH of several members of the pathway and the fact that the Slt2 MAP kinase becomes phosphorylated (and hence activated) upon alkalinization [20]. The authors found 602 genes induced at least two-fold after 15 min and 178 genes after 30 min. These included the *ENA1* ATPase, *KHA1*, genes involved in iron homeostasis, although they were mainly related to siderophore utilization (*FIT1*, *FIT2*, *FIT3*, *ARN1*) and a limited set of genes related to phosphate utilization (*PHO89* and *VTC3*, but not *PHO84*). The number of induced genes involved in glycogen and trehalose metabolism markedly increased in this study. In the case of glycogen, not only enzymes directly involved in glycogen metabolism, such as *GLG1*, *GSY1*, *GSY2*, *GDB1*, and *GLC3*, but also regulatory components of the system, such as the glycogen-related subunits of the type 1 protein phosphatase Glc7 (*GLC8*, *GIP2*, and *PIG2*) were induced. As far as trehalose metabolism was concerned, *TPS1*, *TPS2*, *TPS3*, *TSL1*, and *ATH1* were found induced. Several hexose transporters, including *HXT2*, increased markedly their expression in response to high pH. None of the above genes was dependent on the presence of the Slt2 kinase. Interestingly, several genes encoding cell wall-related proteins, such as *DFG5*, *SKT5*, *GSC2*, and *CRH1*, were induced, at least in part, in a Slt2-dependent way, thus adding the CWI pathway to the mechanisms controlling the transcriptional response to alkaline pH. These genes were not found induced in experiments performed at pH below 8.0 [11,18], suggesting that more extreme alkalinization is needed to trigger this pathway.

The observation that mutations that activate the PKA pathway (such as *ira1 ira2*, and *bcy1*) cause sensitivity to alkaline pH, whereas its deactivation increases tolerance to this stress, prompted Casado and coworkers [21] to examine the role of the PKA pathway in the transcriptional response to high pH. The authors analyzed the response of wild type and *msn2 msn4* cells subjected to pH 8.0 for 10 and 30 min and found that after 10 min of alkaline stress, the expression of many induced genes (47%) was, at least in part, dependent on the presence of the Msn2 and Msn4 transcription factors. The induction of trehalose (*TPS1*, *TPS2*, *TPS3*, *TSL1*, *NTH1*, and *ATH1*) and glycogen (*GLG1*, *GSY1*, *GSY2*, *GDB1*, *GLC3*, and *GIP2*) was almost without exception fully or strongly dependent on the presence of Msn2/Msn4. Genes encoding high-affinity hexose transporters (*HXT2*, *HXT7*, *HXT6*, *HXT4*, and *HXT5*) were induced, but weak or no dependence on Msn2/Msn4 was found. Interestingly, the authors observed that after 30 min, the expression of *ENA1* was accentuated in the *msn2 msn4* mutant, and this effect was attributed to a decrease in the expression of *NRG1* (a repressor of *ENA1* expression) in the *msn2 msn4* strain compared with the wild type. A restricted set of phosphate (*PHM7*, *PHO89*, *SPL2*) or transition metal (*FIT1*) related genes was identified in this work, likely due to the limited time course carried out.

Similarly, the fact that certain mutants in the glucose sensing and response pathways, such as those lacking the AMP-activated Snf1 protein kinase, are sensitive to alkalinization prompted researchers to examine the role of this kinase in the response to alkaline stress [22]. This was done by shifting wild-type and *snf1* cells to pH 8.0 for 10 min. The authors found increased expression of 391 genes (≥2.0-fold threshold), of which 152 (38.9%) displayed some degree of Snf1 dependence. A parallel experiment in which cells were subjected to glucose starvation for 15 min revealed that 75% of the genes induced in the short term by high pH are also induced by glucose scarcity. In concordance with previous works [20,21], the authors identified many genes involved in glycogen metabolism and found that those dependent on Msn2/Msn4 were also dependent on the presence of Snf1. In contrast, the induction of trehalose-related genes, also found to be Msn2/Msn4-dependent in [21], was essentially independent of Snf1. The same set of hexose transports found as induced by Casado and coworkers was found in this work. The expression of *ENA1* and *KHA1* was increased, but only a very limited set of genes related to phosphate (*PHM7* and *PHO89*) and transition metal homeostasis (*TIS11*/*CTH2*) were identified as induced in this work. The paper also reported a decrease in expression of 341 genes, largely enriched in genes involved in ribosome biogenesis (encoding ribosomal as well as rRNA processing proteins), the majority of which were independent of Snf1.

It is worth noting that the transcriptional changes described above do not necessarily derive from the activation (or repression) of the transcription of the affected genes. In fact, Canadell and coworkers [23] performed genomic run-on experiments to determine transcription rate and mRNA levels in cells subjected to a pH 8.0 shift for up to 1 h (with six time points). A major output from this work was the observation that alkalinization causes an overall decrease in transcription rate and a fast destabilization of mRNAs, followed by a more prolonged stabilization phase. In this regard, alkaline stress response is atypical (in comparison to other forms of stress such as oxidative, osmotic, and heat-shock stresses) because of the limited contribution of transcription rate to the increase in mRNA levels. On the other hand, the alkali-triggered decrease in the amount of mRNAs was found to be mainly due to a decline in both the transcription rate and mRNA stability.

The significant number of studies performed in *S. cerevisiae* allows us to draw a general picture of the gene families activated or repressed in response to a short-term (up to 2 h) alkalinization of the medium. This picture is shown in Table 2, in which the genes that suffered significant changes in at least three of the reports discussed above are presented.

### 2.2. Aspergillus nidulans

The ability to tolerate alkaline pH was examined in detail in *A. nidulans*, leading to the identification and characterization of three transcription factors: PacC, required to orchestrate the response to alkaline pH, the calcineurin-dependent transcription factor CrzA, and SltA, important for cation homeostasis [44]. The latter is exclusive to the subphylum *Pezizomycotina*. PacC is processed proteolytically at alkaline pH, and induces the expression of the so-called “alkaline” genes and represses that of the “acidic” ones by direct binding of PacC to specific sequences at target promoters [45].

Picazo and coworkers [24] characterized the transcriptional response to alkalinization in *A. nidulans*, investigating the transition (60 min) of *A. nidulans* to pH 8.0 as well as to 1 M NaCl (sodium stress) in wild-type cells and the *sltA* mutant. These authors found that the expression of 1248 genes was significantly deregulated (|log2 FC| ≥ 1), which represents about 10% of the genome of this organism. Of these, 387 genes were upregulated, and 861 genes were found to be downregulated at pH 8.0. These figures were markedly higher than those obtained by shifting the cells to 1 M NaCl (183 genes upregulated, and 217 genes downregulated), and the comparison of both sets of genes showed that there was a significant difference between the transcriptional responses to alkaline pH and sodium stress (only 43 genes were upregulated, and 74 downregulated under both conditions).

Among the alkali-induced genes, there were *enaA* (*AN6642*) and *enaB* (*AN1628*), encoding sodium ATPases, and two Na^+^/H^+^ antiporters (*AN5035* and *AN4131*), alike to *S. cerevisiae* Nha1 (Table 3). However, a third putative Na^+^/H^+^ antiporter (*AN7250*) was repressed. A Pho89-like Na^+^/Pi transporter (*AN8956*) was also found to be induced, but *AN3781*, encoding a protein similar to Pho89, as well as *AN1612*, coding for a Pho84 ortholog, were repressed. None of these genes was induced by high salt. Interestingly, alkalinization did not elicit an iron-starvation response in *A. nidulans*. Only one gene, *AN7727*, encoding a putative Fre2/3 ferric-chelate reductase, was induced, whereas several genes, including *AN7518* (a Fre4-like ferric reductase), *AN5378* (encoding a Sit1 siderophore transporter), and *AN5397* (a putative multicopper oxidase), were repressed. The authors concluded that the response to alkaline pH implies both SltA-dependent and SltA-independent mechanisms.

### 2.3. Aspergillus fumigatus

A first attempt to define the transcriptome of *A. fumigatus* in response to alkalinization was reported by McDonagh and coworkers [26], together with the profiling resulting from mammalian host-adaptation, in vitro iron depletion, and other conditions. They found 211 genes induced after 1 h of alkaline shift and reported a strong correlation (102 genes) between the genes preferentially expressed during both murine infection and in vitro alkaline adaptation. Although the authors unfortunately did not provide the entire set of data, they reported the activation of the gene encoding a putative vacuolar calcium ATPase (*PMC1*), as well as the H^+^/Pi transporter *PHO89*, an alkaline phosphatase (Afu3g14030), and two genes (Afu6g03690 and Afu4g09440) that likely encode two ENA sodium ATPases. Notably, although they found one plasma membrane zinc ion transporter gene (Afu6g00470) induced, they did not detect any concordance between the profiles of iron starvation and alkaline adaptation.

Loss and coworkers [27] carried out a time-course experiment shifting *A. fumigatus* from pH 5 to pH 8. They found that, in response to alkalinization, 384 genes significantly increased and 126 decreased their mRNA levels at one or more time points. Evaluation of the reported data indicated the effect of alkalinization on phosphate homeostasis was limited. Two of the three genes encoding possible *PHO89* transporters (AFUA_5G14280 and AFUA_3G03010) were induced, whereas AFUA_2G10690, encoding a protein similar to Pho84 and Git1 (glycerophosphoinositol) transporters, was repressed. No other genes related to Pi homeostasis were found differentially expressed. The effect on transition metal homeostasis was also scarce and was restricted to two putative zinc transporters (AFUA_6G00470 and AFUA_7G06570) and a siderophore transporter (AFUA_2G05730). Both genes coding for *A. fumigatus ENA* ATPase genes (AFUA_4G09440 and AFUA_6G03690) were rapidly induced by alkaline pH. An enrichment in genes related to amino acid metabolic processes (essentially Arg, Lys, and aromatic and branched amino acids) was observed. In contrast, genes encoding hexose transporter or enzymes related to glycogen, trehalose metabolism, or the oxidative stress response were essentially absent in the set of induced genes. Analysis of the repressed genes highlighted significant enrichment in genes involved in aerobic respiration and oxidative phosphorylation (such as several subunits of the mitochondrial cytochrome c oxidase complex), as well as in ribosome biogenesis. In the same report, the authors described a parallel experiment challenging the cells with calcium ions (200 mM CaCl_2_). In this case, the impact on the transcriptome was much less intense (135 genes induced and 24 repressed at one or more time points). Interestingly, only 32 genes were differentially affected by both stresses, and often with different directionality or temporal profiles, suggesting that the response elicited by the stresses is scarcely related. In addition, the fact that (i) mutants lacking calcineurin (or its downstream transcription factor CrzA) displayed normal tolerance to high pH; and (ii) the alkaline pH-induced expression of the *ENA1* homolog encoded by AFUA_6G03690 occurs independently of calcineurin and CrzA but requires the presence of PacC led authors to propose that the mechanism of alkaline adaptation in *A. fumigatus* does not depend on calcineurin signaling.

### 2.4. Aspergillus niger

The shift to alkaline pH was studied also in *A. niger* by using RNA-seq technology [46]. The comparisons made were pH 5.0 vs. pH 7.0 and pH 7.0 vs. pH 9.0, and cultures were performed for 3 days (therefore, in contrast with most works found in the scientific literature, these experiments do not examine the short-term response to alkalinization). These authors found that growing the cells at pH 7.0 resulted in a decrease in the expression of ribosome-related genes (66 repressed, none induced) in compassion with those grown at pH 5.0, and this effect was accentuated in the transition from pH 7.0 to 9.0. On the contrary, genes related to DNA replication and mismatch repair, as well as to N-glycan biosynthesis, were enriched among the induced ones. Transporter activity was also affected (both induced and repressed), although specific cases were not mentioned (this paper provided very limited reference to specific genes). Genes related to ncRNA processing and ribosome biogenesis were also enriched among the repressed genes when cells were grown at pH 9.0 in comparison with 7.0. In contrast, induced genes were enriched for carbohydrate pathways, such as pentose phosphate, glycolysis/gluconeogenesis, or galactose metabolism. No specific references to transition metal (iron, copper, or zinc) or phosphate homeostasis are found in this work.

### 2.5. Candida albicans

One of the requirements for *C. albicans* to become pathogenic is the ability to adapt to extracellular pH, including thriving at pH above neutrality [2]. This explains the interest in studying the transcriptional response to alkalinization in this organism and, in particular, the role that Rim101 could play in this response. The interest in the transcription factor was boosted by observations supporting that this pathway is involved in both the pathogenesis and virulence of *C. albicans* [47]. In addition, mutation of *RIM101* leads to hyper-susceptibility to azol-derived drugs as well as to echinocandins [48], implying a role of the Rim pathway in tolerance to antifungal drugs.

In 2004, Bensen and coworkers [28] carried out a genome-wide approach using DNA microarrays and reported 514 alkaline pH-responsive genes, of which 267 were induced by the pH 4.0 to 8.0 transition and 247 were repressed. A significant fraction of these (186) were affected by the pH change and by the lack of *rim101*, indicating a substantial role of the transcription factor in the *C. albicans* alkaline pH response. This study highlighted the induction upon alkalinization of iron, copper, and zinc metabolism-related genes, such as *ARN1*, *FET34*, *FRE9*, *FRP2*, *FTH1*, *CTR1*, *PRA1*, and *ZRT101*, among others (Table 4). These authors reported that many of these genes, particularly those expressed at higher levels at alkaline pH, were, at least in part, Rim101-dependent. This finding was in keeping with the observation that *rim101*^−/−^ mutant strains were sensitive to iron starvation. The induction of *GPX2* and *SOD1* might reflect a possible oxidative stress triggered by alkalinization, although several other genes related to the oxidative stress response were found to be repressed (Table 4).

Alkalinization also affected genes involved in phosphate transport. Thus, *PHO87*, encoding a low-affinity phosphate plasma membrane transporter, was repressed, whereas *PHO84* and *PHO89* were induced (Table 4). Both *PHO87* and *PHO89* expression levels were affected by the lack of Rim101. Other transporters known to be alkaline pH-regulated in *S. cerevisiae* were also found in *C. albicans*, such as *ENA2*, whose induction was also found to be Rim101-dependent. Importantly, this work presented evidence that, in contrast to *S. cerevisiae*, Rim101 does not act through Nrg1 to regulate gene expression but would bind directly to the promoter of the regulated genes.

Another genome-wide approach to understanding the transcriptional response to alkalinization in *C. albicans* was taken by Garnaud and coworkers [29] using RNA-seq technology. These authors tested the response upon transition from pH 4.0 to 7.6 of wild-type (SC5314), *rim101*^−/−^ mutant, and *RIM101*-overexpressing cells. They found 751 genes differentially expressed at pH 7.6 vs. pH 4 for the reference strain (402 upregulated and 349 downregulated genes), although the threshold used was approximately |log2 Fold Change| ≥ 0.59. These figures decrease sharply when a more standard |log2 Fold Change| ≥ 1 is applied (206 upregulated and 142 downregulated genes). Gene Ontology analysis revealed that the alkaline pH-upregulated genes were enriched for genes involved in pathogenesis, whereas downregulated genes were significantly enriched in the transmembrane, drug transport, and metabolic processes. Examination of the data reveals again induction of iron-related genes, such as *FET34*, *FRE7*, *FRE30*, and *FET31*, as well as a potent induction of *ZRT1* (but not of *CTR1*). Genes involved in phosphate acquisition and transport (*PHO100*, *PHO8*, *PHO84*, *PHO89*) and synthesis of polyphosphate (*VTC3*) were also identified as alkaline pH-induced. Interestingly, alkalinization also led to repression of several genes involved in ergosterol biosynthesis (*ERG11*, *ERG1*, and *ERG3*). Comparison of the transcriptional profiles at pH 7.6 for the *rim101*Δ disruption and the SC5314 reference strain yielded 1002 differentially expressed genes (540 upregulated and 462 downregulated). Lack of Rim101 had a large impact on the expression of iron- and zinc-related genes, decreasing the expression of alkaline pH-induced genes and preventing the decrease in the repressed ones. As observed by Bensen and coworkers [28], the absence of the transcription factor also negatively affected the induction of phosphate-related genes, such as *PHO89* and *PHO8*, and resulted in increased expression of *PHO87*. A relevant output of this work was the identification of a couple of Rim101 transcriptional targets, *HSP90* and *IPT1*, as relevant factors in *C. albicans* virulence. Hsp90 is a major molecular chaperone, and Ipt1 is an inositol phosphoryl transferase involved in sphingolipid biosynthesis.

As found earlier for *S. cerevisiae* [19], it has been reported for *C. albicans* [49] that exposure of yeast cells to alkaline stress induces rapid Ca^2+^ influx and a rise in cytoplasmic levels of the cation. The authors also showed that the activation of the *PHO89* promoter under alkaline stress in *C. albicans* was both Crz1- and Rim101-dependent, indicating that this gene is calcium-responsive.

### 2.6. Schizosaccharomyces pombe

Tominaga and coworkers [35] investigated the transcriptional response to alkaline pH in the fission yeast *S. pombe* by studying the transition from pH 5.4 to 8.0 at 2 h after the shift. The authors reported that over 550 genes were induced by at least 2-fold. Although the paper focuses mainly on the analysis of the most potently induced gene (SPBPB21E7.04c, named *cmt*2^+^ by the authors), analysis of the data deposited at NCBI Gene Expression Omnibus (GEO) database (Accession GSE123626) reveals the induction of numerous iron (Str3, Fip1, Fio1, or Str1) and copper (Ctr4, Ctr5, or Ccc2) transporter-encoding genes. However, other transporters, such as Fet4, were repressed. The induction of genes related to iron and copper intake was in line with the previous evidence that disruption of several of these genes resulted in severe inhibition of growth under alkaline pH stress [50]. Two putative Pho84 H^+^/Pi-encoding genes (SPAC23D3.12 and SPCC2H8.02) were also induced, although a third one (SPBC8E4.01c) was repressed. Apparently, there is no homolog for *PHO89* in fission yeast. The data also showed that SPBC839.06 (*cta3* was also moderately induced. Cta3 was initially identified as a Ca^+2^ transporting ATPase but later identified as an ENA ATPase [51], albeit with higher specificity for K^+^ than for Na^+^. Alkalinization also promoted an increase in expression of four hexose transporters and of two genes encoding trehalose synthase, but not that of glycogen-related genes. No evidence for alkali-induced oxidative stress can be extracted from the transcriptomic response. Among the repressed genes, those encoding structural constituents of ribosome and translation were highly enriched, suggesting a halt in protein synthesis.

### 2.7. Debaryomyces hansenii

The response of the halotolerant ascomycete *D. hansenii* to alkalinization was examined by A. Peña’s laboratory using DNA microarrays [36], although it was discussed in the paper mainly within the context of the shift between pH 6 and pH 8 plus 1 M NaCl. Re-analysis of the original data, kindly provided by the authors, reveals that alkalinization alone induces genes related to transmembrane transporter activity, including those involved in phosphate transport, such as DEHA2D01188g (encoding Pho89) and DEHA2E14234g (Pho84), as well as DEHA2D10802g (coding for a presumed Git1 glycerophosphoinositol transporter) and several myo-inositol transporters. Expression of DEHA2G09108g, encoding a likely Na^+^-ENA ATPase, was only modestly increased, likely due to the late (3 h after stress) timing of the sampling. Other relevant subsets of induced genes were two chitin synthases (DEHA2A04642g, DEHA2A08822g), as well as four members of the histone deacetylase complex, including two histone deacetylase enzymes (DEHA2A08712g and DEHA2C16918g). An impact on amino acid metabolisms was also detected, including branched amino acid degradation and His, Arg, and Pro metabolism. Finally, lipid metabolism was affected as well, with increased expression of diverse genes encoding fatty acid metabolism (DEHA2A08646g, DEHA2B13948g, DEHA2B16368g, DEHA2C02178g, and DEHA2D18480g). The set of downregulated genes was mainly enriched in genes involved in ribosome formation and translation, including several ribosomal proteins and translation factors.

### 2.8. Aureobasidium pullulans

*A. pullulans* is a yeast-like ascomycete that belongs to the group known as “black yeast” due to its ability to synthetize melanin during growth. Along its life cycle, *A. pullulans* presents varied cell morphology (yeast-like cells, chlamydospores, and hyphal forms), depending on nutrient availability and culture conditions. This fungus is able to prosper in diverse environments and tolerates a large variety of extreme conditions (polyextremotolerance). Zhang and coworkers [30] investigated the changes in mRNA levels of this fungus growing in acidic (pH 4), neutral (pH 7), and alkaline (pH 10) conditions. These authors found that there were 1109 differentially expressed genes when comparing pH 4.0 vs. pH 7.0 groups (821 genes upregulated and 288 genes downregulated), 2019 for the pH 4.0 vs. pH 10.0 comparison (1352 genes upregulated and 667 downregulated), and 1892 for the pH 7.0 vs. pH 10.0 comparison (926 genes upregulated and 966 downregulated). These values would represent from 8.5 to 17% of the organisms’ genes, according to the figure of 11,844 transcripts reported for the reference strain EXF-150 [52] retrieved from GenBank Project PRJNA207874 (https://www.ncbi.nlm.nih.gov/bioproject/554128), accessed on 12 September 2025, although Zhang and coworkers report 37,383 CDS for the strain HIT-LCY3 that was used for transcriptomic analysis [30]. The transcriptomic data showed that several genes related to hyphal growth and spore formation, as well as genes associated with melanin synthesis, were downregulated by alkalinization. Gene Ontology analysis revealed enrichment in transmembrane transport-related genes at acidic pHs for all comparisons. Similarly, growth at acidic pHs resulted in enrichment in genes related to amino acid metabolism, oligosaccharide biosynthesis, and trehalose biosynthesis. Unfortunately, the paper lacked detailed accompanying information, thus preventing a proper comparison with other fungi.

### 2.9. Trichoderma sp.

*T. virens* is a saprophyte, soil-borne filamentous fungus belonging to the order Hypocreales that is used as a biocontrol agent in controlling plant pathogens. The adaptation to alkalinization of this organism was studied using oligonucleotide microarrays derived from the transcript models of the *T. virens* genome [34]. These authors compared the profile in response to a pH 4.0 to pH 8.0 shift in the wild-type strain, as well as in strains deleted for PacC or expressing constitutively the transcription factor. They found that in the wild-type strain, more than 650 genes were differentially regulated (>2-fold change, *p*-value < 0.05) in response to alkaline pH. This represents about 5% of the >12,000 predicted protein-encoding transcripts. The upregulated gene set contained 321 genes and was enriched in genes related to carbohydrate and inorganic ion transport, as well as metabolism, whereas genes related to transcription, replication, translation, and cell cycle control were underrepresented.

Among the induced transporter-encoding genes ID80915, a putative *PHO89* transporter, zinc and/or iron permeases (ID43455, ID89677, and ID30803), and other enzymes involved in iron acquisition (ID30456 and ID43724) or siderophore biosynthesis (SidA, ID 33825, and 43838) were included. Two genes (ID67662 and ID34827) coding for P-type ATPase Na^+^ transporters similar to *ENA1*, as well as ID10799, encoding a putative Na^+^/H^+^ antiporter similar to *S. cerevisiae* Nha1, were also found to be induced. Among the pH-dependent genes, 157 were differentially expressed under alkaline stress in wild-type cells relative to the Δ*pacC* strain (64 genes downregulated and 93 upregulated), that is, PacC controls, at least in part, about 25% of alkaline pH-regulated genes. Among these were both *ENA1*-like ATPases, the ID 89677 zinc transporter, and the *PHO89*-like gene. Expression of an ortholog of *DFG5*, a cell-wall glycoprotein involved in tolerance to alkaline pH in *Candida albicans*, was strongly upregulated by alkaline pH in the wild-type strain but was not affected by mutation of PacC. Neither Nrg1 nor Smp1, the negative regulators downstream of Rim101/PacC in *S. cerevisiae* [39], were found to be downregulated by alkaline pH. Together with a significant enrichment for PacC sites found within the promoters of PacC-dependent upregulated genes, this suggests that in *T. virens*, PacC could directly bind promoters to mediate its activation upon alkalinization.

*T. reesei* has been considered an important producer of enzymes of commercial interest, such as cellulases and hemicellulases for various industrial applications, as well as used as a host for the production of heterologous proteins [53]. The pH-dependent expression of *T. reesei* genes was investigated by genome-wide transcriptional profiling (using DNA microarrays) and by analyzing the effects of deletion of the gene encoding the transcriptional regulator *pac1*, the ortholog of the *A. nidulans pacC* gene [33]. However, these authors restricted the pHs investigated to pH 3, 4.5, and 6, and samples were collected after 17 and 24 h after the pH shift. Therefore, this work is mentioned here for the sake of completeness but cannot be considered an investigation of the response to alkalinization.

### 2.10. Ustilago maydis

*U. maydis* (renamed as *Mycosarcoma maydis*) is a phytopathogenic fungus belonging to basidiomycetes that raises considerable interest because of its complex life cycle and the ability to infect maize (*Zea mays*). Cervantes-Montelongo and coworkers examined the transcriptional plasticity of this organism in the adaptation to extreme pH by performing DNA microarray-based transcriptomic profiling of cells growing at pH 3, 7, and 9 for 16 h [32]. When *U. maydis* was transferred from neutral pH to pH 9, the authors identified 797 genes differentially expressed, of which 335 were upregulated and 462 downregulated. Functional analysis revealed enrichment in metabolic genes among the induced ones, in transcription-related genes among the repressed ones, and similar enrichment levels in transport-related genes among induced and repressed genes. Two likely *ENA* genes (um11372 and um12056) were induced at pH 9 and, in contrast, an Acu2-related ATPase that mediates high-affinity Na^+^ and K^+^ uptake was strongly repressed. Diverse genes related to the homeostasis of iron (*FER1*, *FER2*, *FER7*, *FER3*, and a probable siderophore transporter (um04410)), zinc (*ZRT2*), and copper (two possible copper transporter-encoding genes, um10210 and um11588) were also induced. The expression of the likely Pho89-encoding gene (um03475) as well as of um04114 (encoding a probable Pho8 repressible vacuolar alkaline phosphatase) was also increased. *VTC1* (um05595) and *VTC4* (um10968) were also induced, albeit with lesser potency. In contrast, no induction of either of the two possible *PHO84* phosphate transporter-encoding genes (um00800 and um06490) was observed. Only one gene encoding a putative sugar transporter (um01656) was found induced, and there was no relevant effect on genes involved in glycogen or trehalose metabolism.

Comparison of the data with the previously reported 1425 genes found differentially regulated by PacC/Rim101 in *U. maydis* [31] revealed that only 292 out of the 797 genes regulated at alkaline pH (36.6%) were in common (160 upregulated and 132 repressed). This suggests that only a minor proportion of the alkali-regulatable genes are under the control of the Pal/Rim pathway and that, therefore, other signaling mechanisms must exist in this fungus.

### 2.11. Komagataella phaffii

*K. phaffii* (formerly known as *Pichia pastoris*) is a methylotrophic yeast widely used for expression of heterologous proteins of industrial interest [54] whose response to environmental alkalinization has been only recently addressed. Albacar and coworkers [37] reported the transcriptional changes for 15, 30, and 60 min after the shift from pH 5.5 to 8.0 and 8.2, in cells growing on glycerol or glucose as carbon source. These authors found 772 DEGs (|log2| FC ≥ 1, *p*-value < 0.01) for at least one time or condition, of which 430 were induced and 378 repressed (36 genes were induced or repressed depending on the condition tested). Most genes induced or repressed in cells growing on glycerol were similarly affected in cells growing on glucose. The transcriptional response after shifting cells to pH 8.0 was very fast, with over 170 genes upregulated and nearly 150 genes downregulated after 15 min, although it was somewhat delayed in cells transferred to pH 8.2.

Functional analysis of induced genes revealed an antioxidant response, including genes encoding Cta1 (catalase A), Ahp1 (a peroxiredoxin), Tsa1 (thioredoxin peroxidase), or Grx2 (a glutaredoxin). Such response was confirmed by microscopy and flow cytometry analysis. Phosphate metabolism was also affected by alkalinization of the medium, including genes encoding components of the vacuolar polyphosphate biosynthetic machinery (Vtc1, Vtc2/3, Vtc4), a putative transcription factor Pho4 (C4QZW1), and the Na^+^/Pi co-transporter Pho89 (C4R021), which was one of the genes most potently induced. In contrast, two genes encoding putative plasma membrane phosphate transporters (C4R3N5 and CAR7K5), equivalent to the *S. cerevisiae* Pho84, were repressed, particularly the latter. The expression of genes involved in iron, copper, and zinc metabolism was altered as well. This included induction of three different Arn1–3 like siderophore transporters (C4R573, C4R468, and C4QZD7), two putative members of the ferric and cupric reductase system (Fre1 and Fre2), the low-affinity Fe^2+^ transporter Fet4, and C4R7J6, which encodes the closest relative to *S. cerevisiae* Cth1 and Cth2. The genes coding for the plasma membrane high-affinity copper transporter Ctr1 (C4R733) and the high-affinity zinc transporter (C4R4S9, Ztr1) were also induced. Interestingly, genes encoding the copper-dependent high-affinity iron import complex Fet3/Ftr1, as well as a putative homolog of Fre3, one of the cell-surface metalloreductases that cooperates with Fet3/Ftr1, were clearly repressed. The different behavior between the non-reductive and reductive uptake systems was attributed to the concurrent condition of oxidative stress. Other relevant functional gene families found induced were those related to thiamine biosynthesis, as well as to the biosynthesis of arginine and sulfur-containing amino acids. On the other hand, functional analysis reveals that in *K phaffii*, alkalinization causes a widespread transcriptional repression of respiratory metabolism, including components of the electronic transport chain, as well as of many genes encoding glycolytic and gluconeogenic enzymes.

Very recently, a second transcriptional study was reported [38] in which the role of two transcription factors likely involved in the alkaline response, Crz1 and Rim101, was investigated. To this end, the authors identified the possible *K. phaffii* candidates (C4R2J3 for Crz1 and C4R535 for Rim101), generated single and double mutant strains, and performed a phenotypic characterization. The transcriptional analysis (shift to pH 8.0 for 15, 30, and 60 min) revealed 772 DEGs (396 genes induced and 340 repressed at least at one time point), with a temporal and functional profile similar to the one described above. Mutation of *RIM101* alone affected about 50% of DEGs, whereas the expression of 6% of these was dependent only on Crz1. Alkali-regulated expression of 38 genes (5%) was affected by both mutations, whereas for 282 genes (39%), the transcriptional changes were not quantitatively altered by any of the mutations, indicating that they are sensitive to alternative alkali-sensitive pathways. Therefore, in comparison with Crz1, Rim101 plays a major role in the alkaline transcriptional response in *K. phaffii*, in agreement with the strong negative effect of the *rim101* mutation on high pH tolerance [38]. The effect of the *crz1* mutation was more noticeable at earlier time points (15 min), whereas the lack of Rim101 was more prominent at 30 and 60 min. The main impact of the *crz1* mutation was a decrease in mRNA production, suggesting a major role in gene induction in response to stress, whereas the lack of Rim101 produced mixed effects: at short time, there was a dominance of induced genes; at 30 min, downregulation prevailed, and at 60 min, positive and negative effects were almost balanced. Mutation of *CRZ1* decreased the expression of the *PMC1* vacuolar Ca^2+^-ATPase, whereas lack of *RIM101* affected the *ENA2* Na^+^-ATPase, the *PHO89* and *PHO84* phosphate transporters, and diverse genes involved in iron homeostasis. Notably, alkalinization led to a repression of the C4R618 gene, encoding the Nrg1 transcriptional repressor (also observed in reference [37]), and this effect was abolished in the *rim101* mutant. This and other evidence led the authors to propose that, as it occurs in *S. cerevisiae*, Nrg1 would mediate the role of Rim101 in alkaline tolerance in *K. phaffii*.

## 3. An Integrative Overview of the Transcriptional Responses to Alkaline pH

The fact that the different reports reviewed in this work are not homogenous, either in terms of the quantitative shift from acidic to alkaline pH or in the sampling timing after the shift is applied, complicates the task of extracting general rules applicable to any fungus. Still, there are several traits that consistently appear and that could be taken as landmarks of the fungal transcriptional response to alkalinization.

**Figure 1:**
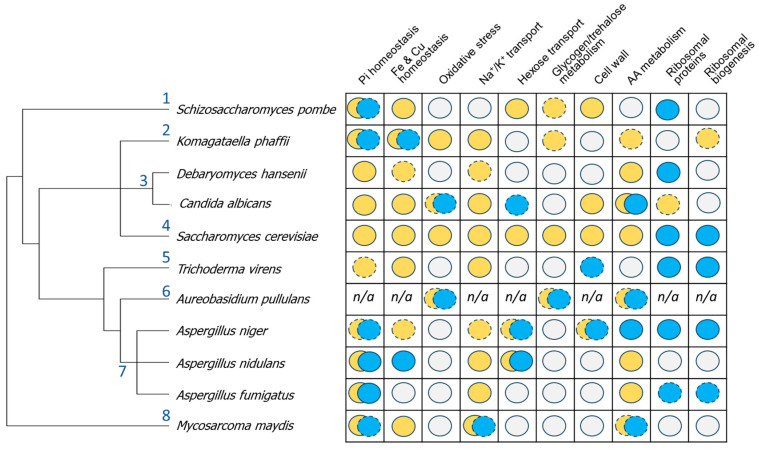
Integrative overview of the transcriptional response of relevant fungi included in this work. Functional families are indicated at the top. Orange circles denote induced expression, blue circles indicate repressed expression, and grey circles represent function not altered under the conditions studied in each case. Discontinuous borders indicate that the effects were weak or not generalized. n/a, information not available. The information for *A. pullulans* corresponds to the pH 4 to pH 10 transition, and for *A. niger*, to the combination of pH 5 to 7 and pH 7 to 9 shifts. The phylogenetic tree was constructed using the R package rotl v. 3.1.0 [65,66]. Numbers represent the following families: 1, *Schizosaccharomycetaceae*; 2, *Pichiaceae*; 3, *Debaryomycetaceae*; 4, *Saccharomycetaceae*; 5, *Hypocreaceae*; 6, *Saccotheciaceae*; 7, *Aspergillaceae*; 8, *Ustilaginaceae*. AA, amino acid.

A rather general alteration that can be observed in the species analyzed is a positive response involving iron (and often related metals such as copper and zinc) homeostasis-related genes (Figure 1). This response likely derives from the decreased bioavailability of these ions when pH increases and from the requirement for copper to sustain iron uptake [55,56]. In fact, it was demonstrated that the availability of iron and copper is a major limiting factor for growth of *S. cerevisiae* at alkaline pH [42]. Efficient acquisition of iron and copper is crucial for pathogenic fungi engaged in systemic infection, when they often encounter pH above neutrality. In this regard, mutations in genes involved in the reductive iron uptake systems decrease virulence in *C. glabrata* and *C. albicans* (see ref. [57] and references therein). It is worth noting that *A. nidulans* and *A. fumigatus* do not respond to alkalinization with increased expression of this subset of genes. This might be due to the fact that these fungi mostly rely on siderophore-mediated uptake [58,59].

Because of the relevance of phosphate for the synthesis of key biomolecules, such as nucleic acids or ATP, maintenance of phosphate homeostasis is crucial for normal growth [60]. It is well known that alteration of the expression of genes related to phosphate acquisition and homeostasis is a common feature in most fungi in response to environmental alkalinization (Figure 1). This is remarkable because the downstream mechanisms for gene regulation in response to Pi starvation differ among fungi. For instance, in *S. cerevisiae* the helix-loop-helix transcription factor Pho4 is responsible for activation of the *PHO* regulon, accounting for about 20–30 genes [12,61], but it requires the participation of the Pho2 co-regulator. However, in *C. albicans*, the participation of Pho2 is not required [62], and in fission yeast, the response to phosphate starvation is mediated by Pho7, a Zn2Cys6 binuclear cluster-containing transcription factor (that is, structurally different from Pho4), which acts as a general stress-responsive factor [63]. The latter variations coincide with an expanded repertoire of gene targets [64].

Overall, alkalinization triggers a response like that produced after phosphate deprivation (Figure 1). A common feature is the induction of the Na^+^/Pi *PHO89* transporter, which is observed almost without exception in all fungi examined (except in *S. pombe*, where the gene likely does not exist). Interestingly, the H^+^/Pi high-affinity transporter *PHO84* is only induced in a subset of fungi (*S. cerevisiae*, *C. albicans*, and *D. hansenii*), whereas in the rest, either its expression does not change or, as happens in *A. nidulans*, *A. fumigatus*, *S. pombe*, or *K. phaffii*, at least one of the isoforms is repressed. In *S. cerevisiae*, *C. albicans*, *U. maydis*, and *K. phaffii*, the induction of all or some of the components of the VTC polyphosphate polymerase complex is observed. In any case, induction of the *PHO* regulon (or part of it) occurs at alkaline pH even in the presence of plenty of phosphate. Most fungi are best suited to grow at acidic external pH, maintained by the pumping of protons across the plasma membrane by the H^+^-ATPase, and disruption of this proton gradient by alkalinization compromises uptake of phosphate (and other nutrients), which is achieved under acidic pH conditions by low- or high-affinity (such as Pho84) H^+^/Pi symporters. Above neutrality, the Na^+^/Pi Pho89 transporter becomes important for growth, as demonstrated in *S. cerevisiae* [67] and *C. albicans* [68], which would explain the induction of the gene observed in most fungi upon alkalinization. In the case of *C. albicans*, expression of Pho89 might contribute to its ability to colonize human niches with neutral or mildly alkaline pH [69]. In this context, it is tempting to speculate that induction of *PHO84* only occurs in those fungi in which the transporter is effective even near neutrality. Thus, in *S. cerevisiae*, Pho84 has been shown to function up to pH 8 [67], whereas in *C. albicans*, it is able to sustain growth even at pH 7 [68]. In other fungi, the presence of Pho84 at the plasma membrane above neutrality might be even detrimental, thus explaining the repression of the gene(s) encoding the transporter.

A common feature in most fungi is the induction of the Na^+^ exporting ATPase *ENA1/2* even in the absence of salt stress. This likely reflects the requirement for the activity of ATPase to remove the excess of toxic Na^+^ cations introduced in the cells by Pho89 together with the phosphate anion. This concerted action is reflected in the co-regulated expression of both genes described for *S. cerevisiae* [70]. It is remarkable that *S. pombe* appears to be an exception to this rule. Two features of this fission yeast might provide an explanation for such discrepancy. From one side, it does not appear to encode a *PHO89* Na^+^/Pi uptake system, thus segregating Pi uptake from sodium influx. On the other hand, whereas *S. pombe* encodes an ENA-like ATPase (Cta3), this is more specific for potassium than for sodium [51], and detoxification of sodium is carried out by a Na^+^/H^+^ antiporter encoded by *sod2* [71,72].

Finally, genes encoding ribosomal proteins and involved in ribosome biogenesis are repressed in many, but not all, of the fungi examined here, whereas repression of respiratory and OXPHOS genes was described for *S. cerevisiae*, *K. phaffii*, and *A. fumigatus*, but not for other fungi. These differences can be attributed, in some cases, to the intensity of the stress applied.

## 4. Linking the Alkaline Transcriptional Response to Biotechnological Applications

The transcriptional response to stress conditions has attracted the interest of scientists for a long time [73], and in recent years, significant advances have been made to harness stress-responsive promoters for effective protein production. For example, Xiong and coworkers [74] showed in *S. cerevisiae* that the p*HSP12* and *pHSP26* promoters were able to efficiently produce yEGFP in response to both high temperature and acetic acid stress. In *P. pastoris*, the use of the *PHO89* promoter to produce both a lipase [75] as well as bacterial and fungal phytases [76] in response to phosphate starvation was demonstrated. More recently, Bernat-Camps and coworkers took advantage of the induction of the *K. phaffii HSP12* promoter under pseudo-starving conditions to efficiently produce lipase B (CalB) from *Candida antarctica* [77]. These authors also showed that application of an osmotic shock did not enhance further production of the enzyme. Very recently, a systematic semi-rational block-scanning approach was applied to this promoter to generate variants that, upon further refining, resulted in a 2-fold increase in extracellular CalB titer in comparison with the native promoter [78].

The relevance of environmental pH in developing microbial cell factories has been reviewed from different points of view [79], and some effort has been made in *S. cerevisiae* in engineering a pH-dependent response to several yeast promoters, such as *YGP1* and *CCW14*, to improve production in low-pH fermentations [80]. However, the interest in exploiting the potential of alkali-regulated yeast promoters for biotechnological applications has only recently emerged. The transcriptomic analysis of the alkaline response in *K. phaffii* reported by Albacar and coworkers [37] highlighted the fact that several promoters inducible by alkalinization could reach mRNA levels close to those of strong constitutive promoters, such as *GAP*, *ENO1*, and *PGK1* or even *TEF1*. This led the same authors to engineer *K. phaffii* strains that expressed a secretable phytase enzyme from different high pH-regulatable native promoters, such as *TSA1*, *HSP12*, and *PHO89*. It was shown for p*PHO89* that expression could be induced by the simple addition of KOH to the medium, and that the expression levels could be modulated by adjusting the pH (from 7.4 to 8.2). It was also found that limiting the amount of phosphate in the medium resulted in almost a three-fold increase in phytase production. By applying a post-transformational vector strategy in liquid cultures, the authors were able to develop p*PHO89*-phytase based strains that were able to surpass the production in shake flasks of a commercial *AOX1*-based strain.

The concept of building synthetic promoters with novel properties, often based on combinatorial strategies, has been developed in the last few years [81]. Very recently, this concept has been applied in *S. cerevisiae* to the use of alkaline pH *cis* regulatory modules. Zekhnini and coworkers [82], starting from a basal p*CYC1*-GFP vector, exploited a combinatorial approach to generate libraries joining Pho4-binding elements extracted from the *PHO84* promoter and 4x tandems of the Crz1-binding site (4x-CDRE) derived from the *FKS2* gene [83]. The most productive clones were selected by flow cytometry and cell sorting to yield over 30 different variants that were further characterized. The stronger combinations were improved by upstream cloning of an STP/MIG region, derived from the *ENA1* promoter, which contains a Stp1/Stp2 site recently identified as alkali-responsive [84]. Four of these constructs were then tested for the expression of a secretable laccase, an enzyme used in the degradation of kraft lignins from biomass. It was found that strong expression, surpassing that obtained from the *GAL1* promoter in cells growing at acidic pH on galactose, could be achieved even at pH 7.6, with minor effects on cell growth rate.

These results suggest that alkaline pH-inducible promoters could be an excellent tool for production of industrially relevant proteins in yeasts. For instance, they would circumvent the need for methanol in the production process in the case of *K. phaffii*. Indeed, the hybrid promoter strategy tested in *S. cerevisiae* for alkaline pH-regulated elements could be likely extended to other fungi of interest. However, a deep understanding of the alkaline transcriptional response and its underlying signaling mechanisms in each specific fungus is needed to sustain a knowledge-guided approach to develop these expression platforms.

## 5. Conclusions

We show here that the transcriptional response to alkalinization implies, in all cases examined, an extensive remodeling of gene expression, affecting between around 500 and 1000 genes, which correspond to about 5–10% of the entire fungal genome. This represents a substantial number of genes, higher than that observed for other stressful conditions, such as high salt concentrations, and likely reflects a widespread impact of alkalinization on fungal physiology. However, a few important points must be considered when attempting to establish comparisons among the different fungi.

**Figure 2:**
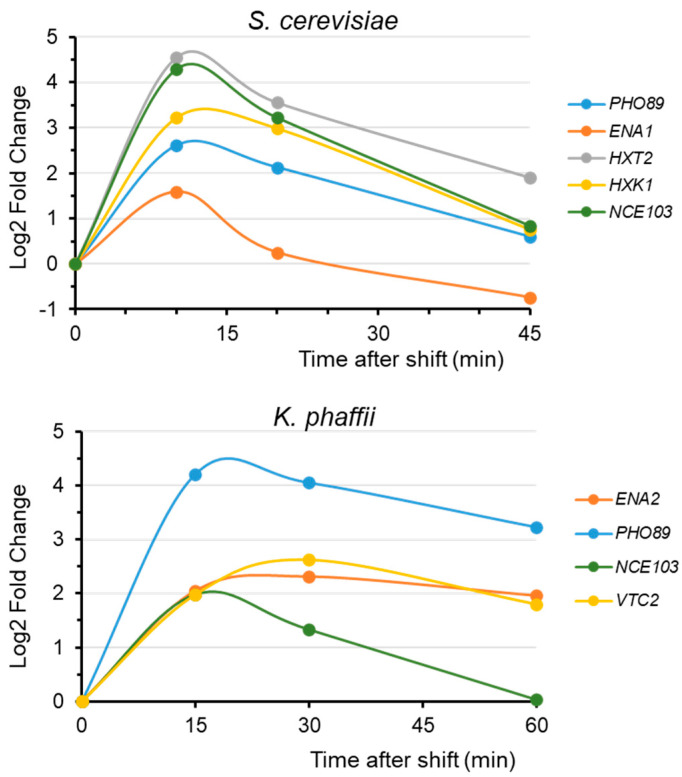
A few examples of time-dependent responses to alkalinization. Data for *S. cerevisiae* were obtained in the author’s laboratory and data for *K. phaffii* were extracted from the Gene Expression Omnibus (GEO) database (Acc. # GSE297128).

From one side, the transcriptional response of many genes to alkalinization is transient. This implies that proper comparisons can only be made for data taken at reasonably similar times after the onset of stress. Figure 2 provides several examples taken from *S. cerevisiae* and *K. phaffii* and shows that the identification of a gene as alkaline pH-regulated (or the quantification of the effect) very much depends on when sampling is performed.

On the other hand, the intensity of stress also influences the panel of genes transcriptionally affected. *S. cerevisiae* offers several examples: (i) the exposure to even mild alkalinization (<pH 8.0) triggers the induction of genes related to transition metal and phosphate homeostasis, but not that of hexose transporters or glycogen and trehalose metabolism; (ii) consistent induction of cell wall protein-encoding genes and activation of the Slt2 kinase were only detected at more extreme pH (8.2); and (iii) mild alkalinization does not invoke repression of ribosomal biogenesis-related genes, as it occurs when shifting cells to higher pH values. The influence of the magnitude of pH change can be also observed in *C. albicans*, where cells shifted from pH 4.0 to 8.0 show repression of genes involved in amino acid metabolism [28], whereas this effect was not reported in a subsequent study when cells were exposed to pH 7.6 [29].

Finally, a more effective comparison of the responses across the diverse fungi examined in this review was hampered by the fact that primary data were often not readily available. In some cases, such fundamental data were not offered as Supplementary Material with the publication but deposited in local repositories no longer accessible, whereas in other cases, the data simply were never made public. A warning note should be issued to the journal’s editors and referees to ensure that, prior to acceptance of a paper, the original data associated with large-scale studies are easily and permanently accessible to the public.

## Figures and Tables

**Table 2:** Typical functional families and genes whose expression is affected by alkalinization in *S. cerevisiae*.

Induced Genes
Phosphate transport and metabolism
*PHO89*, *PHO84*, *PHO12*, *VTC1*, *VTC3*, *VTC4*, *SPL2*, *PHM7*
Iron and copper homeostasis
*FRE1*, *FET3*, *FET5*, *FIT1*, *FIT2*, *FIT3*, *ARN1*, *ARN2*, *TIS11*
Oxidative stress response
*NCE103*, *GRX1*, *GRX6*, *TSA2*, *GRE3*
Glycogen metabolism
*GSY1*, *GSY2*, *GLC3*, *GDB1*, *GLG1*, *GIP2*, *GLC8*, *PIG2*
Trehalose metabolism
*TPS1*, *TPS2*, *TPS3*, *TSL1*, *ATH1*, *NTH1*
Sugar transport
*HXT2*, *HXT7*, *HXT6*, *HXT4*, *HXT5*
Na^+^/K^+^ transport
*ENA1*, *ENA2*, *KHA1*
Cell wall
*SRL3*, *DFG5*, *SKT5* ^(1)^, *GSC2*, *CRH1*
Acetate synthesis
*ALD3*, *ALD2*, *ALD4*, *ALD6*, *ACS1*
Amino acid metabolism ^(2)^
Arg biosynthesis (*ARG4*, *ARG3*, *ALT1*, *ARG7*, *ARG1*)
Cys and Met metabolism (*SER3*, *STR3*, *SER33*, *ADI1*, *SER1*)
His metabolism (*HIS4*, *HIS5*, *ALD3*, *ALD2*)
**Repressed genes**
Ribosomal proteins
*RPS22A*, *RPL14A*, *RPS25B*, *RPL42A*, *RPL25*, *RPL33B*, *RPS10A*, *RPS9A*, *RPL19A*, *RPS16B*, *RPL12B*, *RPL27B*, *RPS23A*, *RPL42B*
Ribosomal biogenesis
*JJJ1*, *RRB1*, *RPB95*, *DBP10*, *ARB1*, *NOC2*, *BMT5*, *PWP1*, *SDA1*, *KRI1*, *RRS1*, *ZUO1*, *NSR1*

^(1)^ Only found in ref. [20]. ^(2)^ Several genes are repressed in ref. [18].

**Table 3:** Typical functional families and genes whose expression is affected by alkalinization in *A. nidulans*. Extracted by re-analysis of supplemental data from ref. [24]. The names of the putative orthologs in *S. cerevisiae* are provided for phosphate-related genes.

Induced Genes
Phosphate transport and metabolism
*AN8956 (PHO89)*
Iron and copper homeostasis
*AN3690*, *AN8365*, *AN7518*, *AN5397*, *AN5378*, *AN3763*, *AN3264*, *AN0878*
Amino acid metabolism
*AN4355*, *AN10298*, *AN5426*, *AN5610*, *AN1673*, *AN0840*, *AN4401*, *AN1883*, *AN6231*, *AN5701*, *AN5886*, *AN8866*, *AN7722*, *AN0717*, *AN5957*, *AN1990*, *AN5999*, *AN2914*, *AN2873*, *AN6782*
tRNA aminoacylation
*AN9157*, *AN7479*, *AN1913*, *AN5662*, *AN0705*, *AN10195*, *AN10475*, *AN0057*, *AN1380*, *AN11125*, *AN2150*, *AN4550*
Glucan and chitin metabolism
*AN2388*, *AN3046*, *AN8480*, *AN0558*, *AN9380*, *AN1602*
Na^+^/K^+^ transport
*AN1628*, *AN6642*, *AN5035*, *AN4131*
**Repressed genes**
Phosphate transport and metabolism
*AN1612 (PHO84)*, *AN5935 (PHO84)*, *AN5549 (GIT1)*, *AN1148 (PPN1)*, *AN8363 (PHO11)*
Fatty acid biosynthesis
*AN8412*, *AN1034*, *AN7855*, *AN4135*, *AN7856*, *AN2035*, *AN2032*, *AN3612*, *AN6731*, *AN3396*, *AN0981*, *AN0918*, *AN7825*, *AN0523*, *AN3386*, *AN3282*, *AN9407*, *AN10430*, *AN1036*, *AN6126*, *AN3276*, *AN3381*
Na^+^/K^+^ transport
*AN7250*, *AN1022*

**Table 4:** Typical functional families and genes whose expression is affected by alkalinization in *C. albicans*.

Induced Genes
Phosphate transport and metabolism
*PHO84 (C1_11480W_A)*, *PHO89 (C4_01940W_A)*, ***PHO8 (C1_10430W_A)***, ***PHO100 (C1_07430W_A)***, *GDE1 (C5_04510W_A)*, ***PHO4 (C4_05680W_A)***, ***VTC3 (CR_03610C_A)***
Iron, copper, and zinc homeostasis
*FRE7 (CR_07290W_A)*, ***FRE30 (CR_07280W_A)***, *SIT1 (C2_08050C_A)*, *FET34 (C6_00440C_A)*, ***FET31 (C6_00480C_A)***, *FRE9 (C2_05070W_A)*, *SMF3 (C2_00580C_A)*, *SMF12 (C2_07160W_A)*, *FRP2 (C7_00100W_A)*, *CFL1 (C4_05770C_A)*, *CFL2 (C4_05780C_A)*, *FTH1 (C1_09400C_A)*, *FLC1 (C3_00980W_A)*, *FTR1/2 (C1_14220C_A)*, *CTR1 (C6_00790C_A)*, *ZRT1 (C4_06970C_A)*, ***CCC2 (C5_03020W_A)***
Na^+^/K^+^ transport
*ENA2 (C1_00390W_A)*, ***ENA21 (C7_02910W_A)***, *C3_01680C_A*
Antioxidant activity
*GPX2 (C6_00840W_A)*, *SOD4 (C2_00660C_A)*, ***SOD5 (C2_00680C_A)***
Cell wall and hyphal growth
*ALS10 (orf19.2355)*, *ALS1 (C6_03700W_A)*, *CHT2 (C5_04130C_A)*, *CRH1 (C4_02900C_A)*, *ECM38 (C4_03540C_A)*, *KRE6 (C3_05830W_A)*, *PHR1 (C4_04530C_A)*, *PRA1 (C4_06980W_A)*, *SRB1 (C3_07950C_A)*, *UAP1 (C5_02530W_A)*, *HYR1 (C1_13450W_A)*, *ECM21 (C1_10180C_A)*, *MP65 (C2_10030C_A)*, *PGA31 (C4_04080C_A)*, *CSA1 (C7_00090C_A)*, *ECE1 (C4_03470C_A)*, *HWP1 (C4_03570W_A)*, *HYR1 (C1_13450W_A)*, *IHD1 (C6_03850C_A)*, *RBT1 (C4_03520C_A)*, *RBT4 (C1_07030C_A)*, *SAP4 (C6_03500C_A)*, *SAP6 (C6_02710C_A)*, *SAP9 (C3_03870C_A)*, ***SAP5 (C6_03030W_A)***
Ribosomal proteins
*RPL10A (C6_02240C_A)*, *RPL11 (C2_06810C_A)*, *RPL13 (C1_03020C_A)*, *RPL15A (CR_04100C_A)*, *RPL16A (C1_00180W_A)*, *RPL18 (C3_05100C_A)*, *RPL21A (C2_03810C_A)*, *RPL23A (C6_02070C_A)*, *RPL3 (C2_09430W_A)*, *RPL4B (C1_14110C_A)*, *RPL5 (C7_01790C_A)*, *RPL8B (C3_05240C_A)*, *RPL9B (C3_02470C_A)*, *RPS1 (C1_03090W_A)*, *RPS12 (C3_07150C_A)*, *RPS15 (C3_04670C_A)*, *RPS18 (C7_00960W_A)*, *RPS20 (CR_08150W_A)*, *RPS22A (C1_06460C_A)*, *RPS26A (C2_01610C_A)*, *RPS4A (C2_10620W_A)*, *RPS6A (C4_01270W_A)*, *RPS7A (C3_01490W_A)*, *RPS8A (C2_05610C_A)*
Amino acid metabolism
*AAT1 (C2_05250C_A)*, *ARO4 (C1_05110C_A)*, *ARO7 (C1_11500C_A)*, *ARO8 (C2_00340C_A)*, *CAR1 (C5_04490C_A)*, *CAR2 (C4_00160C_A)*, *CYS3 (CR_08340W_A)*, *CYS4 (C1_01870C_A)*, *IDP1 (C2_05890C_A)*, *ILV6 (C4_01370W_A)*, *LEU3 (C5_02180C_A)*, *PRO3 (C4_00240C_A)*, *PUT1 (C5_02600W_A)*, *PUT2 (C5_04880C_A)*, *SAH1 (C5_04270C_A)*, *SAM2 (C1_11450C_A)*, *SAM4 (C1_08410C_A)*, *MET15 (C4_00200C_A)*
Amino acid transport
*GAP6 (C5_03500W_A)*, *GAP1 (C5_02790C_A)*, *HIP1 (C5_01800C_A)*, *GNP2 (CR_09920W_A)*, *MUP1 (C1_11870W_A)*, *AGP2 (C4_01100C_A)*
**Repressed Genes**
Iron, copper, and zinc homeostasis
*CCC1 (C3_03710W_A)*, *CRP1 (C1_09250W_A)*, *FRE10 (C4_04320W_A)*, *ZRT2 (C2_02590W_A)*, *CFL4 (C5_01360W_A)*, ***FET3 (C6_00460C_A)***, ***CTR2 (C1_08620W_A)***
Sugar transport
*HGT17 (C4_01070W_A)*, *HGT19 (C3_00220W_A)*, HGT13 (C7_00290C_A) *, *HXT5 (CR_03450W)*, ***HGT10 (C6_03790C_A)***
Amino acid metabolism
*ACO1 (CR_08210C_A)*, *ARG1 (CR_00620C_A)*, *ARG3 (C6_03230W_A)*, *ARG4 (C7_03570W_A)*, *ARG5*, *6 (C1_09290C_A)*, *ARO10 (CR_06860C_A)*, *CHA1 (C2_01270W_A)*, *CPA1 (C4_01550C_A)*, *GAD1 (C1_11660W_A)*, *GLT1 (C1_06550W_A)*, *LEU1 (CR_00360C_A)*, ***CAN1 (C6_00960W_A)***, ***LEU4 (C1_00170W_A)***, ***MET16 (C4_07030W_A)***, ***GAP2 (C3_05580C_A)***
Antioxidant activity
*GTT12 (C3_03600C_A)*, *SOD2 (C1_01520C_A)*, *DOT5 (C3_00480C_A)*, *TTR1 (C1_00490C_A)*, *AHP1 (C4_02410C_A)*, *CCP1 (C3_02480C_A)*, *CAT1 (C1_06810W_A)*
Electron transfer
*SDH2 (CR_05180C_A)*, *NDH51 (C2_04550C_A)*, *FESUR1 (C3_07060W_A)*, *RIP1 (C3_04430W_A)*, *C2_07550W_A*, *NUC2 (C7_01900W_A)*, *CYT1 (C2_04950C_A)*, *TTR1 (C1_00490C_A)*, *CYC1 (C2_10110W_A)*, *C5_01960C_A*

* Upregulated in Garnaud et al. [29]. The systematic names have been added by the author and were obtained from the Candida Genome Database (http://www.candidagenome.org/), accessed on 13 November 2025 The table integrates data from refs. [28,29]. Bold type indicates genes reported only in ref. [29].

## Data Availability

No data was created in this work.
